# A Low-Risk HPV-Associated Well-Differentiated Squamous Cell Carcinoma of the Cervix with Low-Grade Squamous Intraepithelial Lesion Morphology: Clinical and Pathologic Diagnostic Difficulties and Review of the Literature

**DOI:** 10.5146/tjpath.2024.13189

**Published:** 2024-09-02

**Authors:** Deniz Ates, Esra Nur Sahin, Kübra Katipoglu, Alp Usubutun

**Affiliations:** Department of Pathology, Hacettepe University, Faculty of Medicine, Ankara, Türkiye; Clinics of Pathology, Diskapi Research and Educational Hospital, Ankara, Türkiye; Department of Pathology, University of Health Sciences, Ankara Bilkent City Hospital, Ankara, Türkiye

**Keywords:** Cervix, Condylomatous carcinoma, HPV, Koilocytosis, Squamous cell carcinoma

## Abstract

Approximately 95% of cervical squamous cell carcinomas are associated with high-risk HPV, with a small number of HPV-independent tumors. However, low-risk HPV types have also been detected in rare cervical squamous cell carcinomas. Low-grade squamous intraepithelial lesion-related changes are a rare morphologic finding in cervical squamous cell carcinoma. We present the case of a 30-yr-old woman who presented with pelvic pain and foul-smelling vaginal discharge showing an exophytic lesion protruding from the cervix. Repeated superficial biopsies showed a low-grade squamous intraepithelial lesion (LSIL) characterized by binucleation and koilocytosis. Chromogenic in-situ hybridization revealed the presence of HPV6/11. The absence of high-risk HPV was confirmed by PCR. After following the patient for nine months without intervention, type III hysterectomy and bilateral pelvic paraaortic lymphadenectomy were performed. Microscopic examination showed well-differentiated squamous cell carcinoma with solid epithelial islands and extensive eosinophilic cytoplasm without pleomorphism. HPV 6 and 11 were also detected with chromogenic in-situ hybridization. Neoplasm invaded the full-thickness of the cervical wall and infiltrated the vagina, parametrium, the proximal ureter and bladder. The patient who received chemoradiotherapy is disease-free at 36 months follow-up. Low-risk HPV-related well-differentiated invasive squamous lesions exist, and such lesions could be a diagnostic pitfall for gynecologists and pathologists; in these cases, radiologic-pathologic correlation and radiologic guided biopsy are mandatory.

## INTRODUCTION

According to data from the International Agency for Research on Cancer (IARC), cervical cancer is the 4th most common cancer in women globally. Around 80-90% of cervical cancers are squamous cell carcinomas. In the most recent World Health Organization classification, cervical squamous cell carcinomas are categorized as either “HPV-associated” or “HPV-independent” (1). Around 95% of cervical squamous cell carcinomas are HPV-associated, and almost all are high-risk HPV types (especially type 16 and 18). However, low-risk HPV types (types 6 and 11) have also been detected in a very small number of cervical squamous cell carcinomas (1–4).

In the literature, some cases are called condylomatous carcinoma similar to the presented case (Table 1). Cases referred to as condylomatous carcinoma in the literature make up a highly heterogeneous group. In these reports, the HPV types and morphologies differ widely. Those with high-risk HPV and low-risk HPV or with both, and with or without poorly differentiated morphology, are named condylomatous (warty) carcinoma (3–8). For this reason, in this presentation we preferred to use the terminology “low-risk HPV-associated well-differentiated squamous cell carcinoma (SCC) of the cervix with koilocytotic morphology” as Lui et al. mentioned in their study (3).

“Low-risk HPV-associated well-differentiated SCC of the cervix with koilocytotic morphology” is a rare variant of cervical squamous cell carcinomas. Some authors also refer to low-risk HPV-associated tumors with exophytic or verruco/papillary growth patterns (2,3). Histopathologic features are characterized by well-differentiated areas with vacuolization and koilocytosis that mimic condylomas. Well-differentiated invasive squamous islands are seen in the stroma. Prognostic data for this variant, especially in the cervix, have been quite limited, with some studies showing a better prognosis (9,10).

This case was deemed worthy of presentation due to the development of cervical cancer with a low-risk HPV infection. This is challenging to recognize both clinically and morphologically. There is debate regarding the terminology, and its pathogenesis remains poorly defined. A clinicopathological-radiologic correlation is essential for the diagnosis.

## CASE REPORT

A 30-year-old woman presented to the obstetrics clinic with complaints of menstrual irregularity, pelvic pain, and foul-smelling vaginal discharge for 3-months. An exophytic cervical papillary lesion completely involving the cervix was observed on examination. Punch biopsies were taken from the patient.

**Figure 1:**
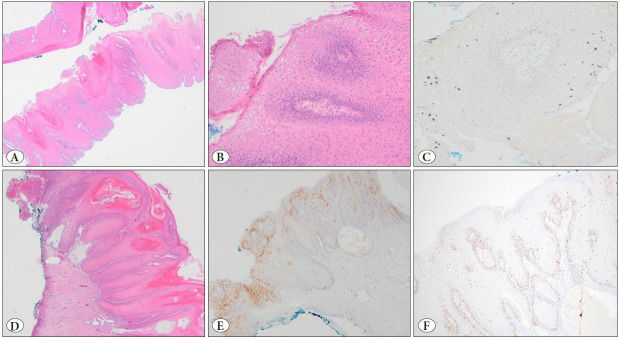
Superficial cervical epithelial fragments with papillomatous architecture at low power (**A,** H&E, 40x). Binucleation and koilocytosis were present in superficial biopsies (**B,** HE, 200x). Low-risk HPV (HPV 6/11) was detected by chromogenic in-situ hybridization (ISH) (**C,** Low-risk HPV-ISH, 200x). Papillomatous hyperplasia and hyperkeratosis were present in the LEEP biopsy (**D,** H&E, 40x). Immunohistochemical studies showed patchy staining with p16 (**E,** p16, 40x) and wild-type staining (patchy in the basal layer) with p53 (**F,** p53, 100x).

On microscopic examination, superficial cervical epithelial fragments were observed (Figure 1). Papillomatous hyperplasia, parakeratosis/hyperkeratosis, and koilocytotic changes were seen on the surface (Figure 1). There was no HSIL morphology. Since the biopsy material was superficial, the subepithelial area was very limited, and no specific pathologic abnormality was observed in this limited stroma. Immunohistochemical studies showed patchy staining with p16 and wild-type staining with p53. Proliferative activity with Ki-67 was limited to the basal epithelial layer. Low-risk HPV (HPV 6/11) was detected by chromogenic in-situ hybridization (Figure 1). According to the described morphological and immunohistochemical findings, the case was diagnosed as LSIL. After the biopsy, the patient was followed clinically and radiologically for 9 months. In the upper and lower abdomen CT of the patient at this period, a lesion originating from the cervix, with air and fluid components in the middle, and infiltrating the outside of the cervix and the lower end of the ureter, which could not be differentiated as benign or malignant, was observed. Due to radiological suspicion and the detection of a persistent lesion on the cervix at the 12-4 o’clock position during the gynecological examination, cervical LEEP was performed. Histopathological examination of the LEEP material revealed a thicker epithelium compared to the first biopsy. Verrucous hyperplasia, broad parakeratosis/hyperkeratosis, and koilocytotic changes were observed on the epithelial surface (Figure 1). The epithelium was devoid of dysmaturation, atypia, and mitosis. Although the subepithelial area was slightly deeper than the first biopsy, no invasion was detected in the subepithelial area. Immunohistochemical studies were also performed on the LEEP material. They showed results similar to the first biopsy (patchy pattern with p16 (Figure 1), wild-type staining with p53 (Figure 1), positivity in the basal epithelial layer with Ki-67). The case was again diagnosed as a low-grade lesion and interpreted as compatible with condyloma. It was reported that although an invasive focus could not be detected, the patient should be followed closely due to the superficial biopsy.

**Figure 2:**
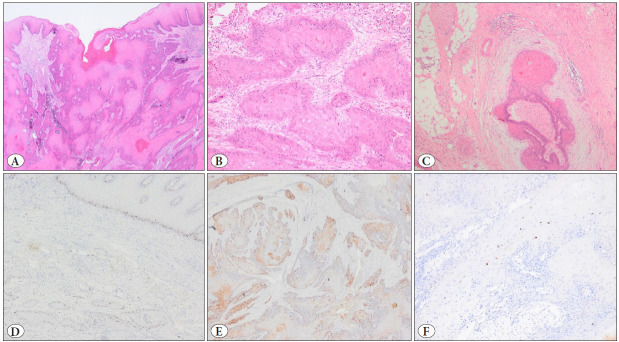
Hysterectomy showed a malignant epithelial tumor that invaded large areas under the epithelium (**A,** H&E, 100x). Welldifferentiated squamous islands with extensive eosinophilic cytoplasm infiltrating the stroma (**B,** H&E, 200x). Deep parametrial and perineural invasions are present (**C,** H&E, 100x). Immunohistochemical studies showed wild-type staining (patchy staining in the basal layer of the infiltrative islands) with p53 (**D,** p53, 100x) and patchy staining with p16 (**E,** p16, 100x). Low-risk HPV (HPV 6/11) was detected by chromogenic in-situ hybridization (ISH) in the invasive squamous islands (**F,** Low-risk HPV-ISH, 200x).

In the lower abdominal MRI performed at the same time as the second biopsy, a soft tissue mass with significant destruction of the cervix and infiltration of the parametrium and the bladder base with necrotic areas was observed. A type III hysterectomy and pelvic paraaortic lymph node dissections were performed. In the macroscopic examination, a mass 4 cm in diameter surrounded the cervical canal and showed deep full-thickness infiltration. Microscopic examination showed a well-differentiated SCC, unlike the previous biopsies (Figure 2). The tumor consisted of well-differentiated solid epithelial islands with extensive eosinophilic cytoplasm and no obvious pleomorphism (Figure 2). Perineural invasion was detected (Figure 2). Immunohistochemical studies were applied with p16 (Clone MX007; Dilution 1/200, Citrate, Acadia, California, USA) and p53 (Clone DO7; Dilution 1/600, Citrate, Leica, Newcastle, UK). HPV probes (Bond HPV 6/11 probe, Leica, Newcastle, UK) for HPV subtypes 6 and 11 were used for low-risk HPV identification by DNA in-situ hybridization. Immunohistochemical examination showed a wild-type reaction with p53 in neoplastic cells (Figure 2). p16 was patchy positive (Figure 2). Low-risk HPV (HPV 6/11) was detected in this biopsy with chromogenic in-situ hybridization (Figure 2).

HPV PCR was also performed for high-risk HPV types. Sections were taken from the tumor containing formalin-fixed and paraffin-embedded block and collected into an RNAse-free reaction tube. The instructions specified with the QIAsymphony DSP Virus/Pathogen Kit, QIAGEN (Strasse 1, 40724 Hilden, GERMANY) were followed and cell lysis was performed. Subsequently protein denaturation occurred by increasing the temperature and adding protein kinase K. DNA precipitate was obtained by centrifugation. In the test applied to evaluate high-risk HPV, the QIAscreen HPV PCR Test was performed on DNA extracted from the patient’s tumor tissue using the QIAsymphony automatic DNA isolation device on the QiaRotor gene platform. In this platform, the lower detection limit is defined as 255 copies for HPV 16, 456 copies for HPV 18 and 56, 4630 copies for HPV 31, 33, 35, 39, 45 51 59 68 and 46307 copies for other HPV types. In our case, the absence of high-risk HPV was confirmed by PCR.

Neoplastic cell clusters invaded the full-thickness of the cervical wall and infiltrated the vagina, parametrium, and proximal ureter and bladder. No metastatic lymph node was detected in the pelvic paraaortic lymph node dissection.

The latest biopsy revealed infiltrative islands that tested negative for p16 by immunohistochemistry and low-risk HPV-ISH positivity. The patient was diagnosed with “well-differentiated SCC developing with low-risk HPV”. Thirty-six months have passed after the patient’s hysterectomy, and our patient has been living without the disease for 36 months. The patient received chemoradiotherapy during this period. The latest MRI scan showed no residual or metastatic disease.

## DISCUSSION

Low-risk HPV-related squamous carcinomas, especially in the cervix are very rare. Some have called these lesions “condylomatous carcinoma” and the information about their histomorphology, immunophenotype, and epidemiological and prognostic data is limited. Case reports in the literature also show different characteristics (3-5 and 7-9). We searched cases with the keyword “condylomatous carcinoma” and those with “condylomatous features” in the English literature and encountered six patients and four articles demonstrating low-risk HPV typing, and reliable and negative p16 results consistent with HPV typing. These findings included hyperplastic/papillomatous epithelium with koilocytotic morphology on the surface, as well as well-differentiated invasive islands in the deep. Details of the cases in the literature and comparison with our case are given in Table 1. The ages of the patients were between 43 and 75 years. Our patient is 30 years old, and she is the youngest patient in the literature. The FIGO stage at the time of diagnosis is available in a small number of cases and is low in those cases.

**Table 1:** Clinicopathological characteristics of the patients were searched with the keyword condylomatous carcinoma in English literature. The patients with low-risk HPV type, negative p16, and binucleation on the surface epithelium and well-differentiated squamous islands, like our case, were shown.

**Cases**	**Age**	**Stage**	**Histopathologic Features** **(According to provided photomicrographs)**	**p16**	**HPV-ISH**	**PCR**
**Masuda et al. **(7) **(1 case)**	43	Not Available	Papillary formation, thick epithelium koilocytosis Prominent stromal invasion (Well differentiated squamous islands in the stroma)	(-)	Not Available	HPV6
**Kim and Lee **(8) **(1 case)**	75	FIGO IB1	Thickened squamous epithelium Koilocytotic atypia, Condylomatous fronds on the top Well-differentiated squamous invasion in the stroma	(-)	Not Available	HPV6, HPV42
**Liu et al. **(3) **(3 cases)**	44, 50, 51	Not Available	1st Case: Biphasic low–grade and high-grade papillary SIL with microinvasion and LN metastasis 2nd Case: Papillary SIL with superficial invasion 3rd case: Fragments of papillary SIL	3 cases are (-)	Not Available	HPV 6
**Rokutan-Kurata** **et al. (Case 3) **(4) **(1 case)**	43	T1b1N1M0	Well differentiated squamous epithelium with papillary and condylomatous growth pattern and stromal invasion	(-)	Not Available	HPV 6
**Our Case**	30	FIGO IVA	Thickened epithelium: Papillomatous hyperplasia Koilocytosis Well differentiated, invasive solid squamous nests	(-)	HPV 6,11 (+)	Not Available

In our case, the disease had progressed to an advanced stage due to the patient’s late diagnosis. Our case highlights that these tumors can invade distant organs even when caused by a low-risk HPV strain. Masuda and colleagues reported a case of HPV 6-associated, rapidly progressive condylomatous carcinoma that was followed for three years by a smear diagnosis of LSIL and showed negative p16 and increased p53 positivity, with the authors speculating that p53 positivity could be related to the aggressive course (7). Our patient was at the FIGO IVa stage at the time of diagnosis, and p53 expression was not widespread; it had a wild-type staining pattern. Diagnostic difficulty was reported in one case, just like the presented case. Rokutan-Kurata M. et al.’s case 3 was diagnosed with LSIL by smear, and it took 2 years to reach a definitive diagnosis (4).

Cases referred to as condylomatous carcinoma in the literature are cases with exophytic protrusions from the cervix; morphologic features and HPV types are heterogeneous. For example, there is a case associated with high-risk HPV (5), a case in which HPV was never detected (6), and a case in which both high-risk and low-risk HPV were detected (11). In addition, the morphologic features are also heterogeneous. For example, in some cases, HSIL morphology secondary to high-risk HPV and poorly differentiated invasive islands dominate (5). In contrast, in some cases, LSIL morphology is characterized by koilocytosis in the surface epithelium and well-differentiated infiltrative islands dominate (3, 7 and 9). Because of this heterogeneity, we decided to name the presented case “low-risk HPV-associated well-differentiated SCC of the cervix with koilocytotic morphology” as Liu et al. mentioned in their study (3). Cases with cervical protrusion, koilocytotic changes, deep well-differentiated invasive islands, and low-risk HPV are summarized in Table 1.

Low-risk HPV-associated cervical cancer is rare. In a recent large series of 670 cases of cervical squamous cell carcinoma, no low-risk HPV-associated SCC was found (12). Incidence of low-risk HPV-associated cancer is less than 1% in large datasets (2,13). To our knowledge, there is no detailed study in the English literature that describes the pathogenesis of low-risk HPV-associated cancers. There has been a brief discussion on this topic. In their study, Dovey de la Cour et al. concluded that “this finding is likely due to co-infection with an unrecognized oncogenic HPV type” (13). According to a retrospective global review of HPV genotyping in invasive cervical cancer, it is uncertain how some HPV types, including HPV 6 or 11, cause cancer. Although these types were infrequently found in cervical SCC, the widely known causality could not be conclusively proven (14). Perhaps for this reason, HPV 6 and 11 were reclassified from group 2B (possibly carcinogenic to humans) to group C (not classifiable as carcinogenic to humans) in 2007 and 2012, respectively, according to the IARC monographs (15,16), and their carcinogenic potential was reduced. However, since the part of our tumor that protrudes from the cervix shows changes indicative of HPV infection, such as koilocytosis and binucleation, we suspect that low-risk HPV does not hang around there and that the tumor is most likely HPV-associated. A recent publication stated that “The genomic similarities between HPV6/11 and HPV16 mainly involved the E7 gene, indicating a limited ability to block cell differentiation” (17).

From the morphologic point of view, there are some issues that must be clarified. Verrucous carcinoma was included in the differential diagnosis due to having both papillomatous/verrucous protrusions on initial biopsies and well-differentiated squamous islands in the radical material. However, low-risk HPV was detected in the malignancy in the presented patient. The HPV results were shown to be negative in nearly all verrucous carcinomas of the anogenital system in the literature when strict histological criteria were applied (18,19). In addition, verrucous carcinoma is unlikely to exhibit binucleation or halo. With the knowledge in conjunction, verrucous carcinoma was ruled out. Additionally, it exhibits morphologic alterations similar to condylomas and LSILs, such as binucleation and koilocytosis.

The follow-up period of the patient is 36 months, and the patient has been living without the disease for 36 months. Recent MRI revealed no findings suggestive of recurrent mass or distant metastasis.

In conclusion, a low-risk HPV-associated well-differentiated cervix SCC with koilocytotic morphology is a tumor that should be included in the differential diagnosis with benign lesions such as condyloma. It provides a diagnostic challenge for both clinicians and pathologists. Keep in mind that in squamous cell carcinomas with low-risk HPV, koilocytic changes and condyloma-like surface features might be found, and the presence or absence of a residual lesion after a biopsy should be investigated. When condyloma-like morphology is detected in an equivocal case, clinic-pathologic and radiologic correlation is required to avoid falling into the diagnostic trap.

## Conflict of Interest

The authors declare that they have no conflict of interest for this article.

## Ethics Approval

Not applicable, anonymity of the patients’ and their confidentiality was preserved.
